# Investigating the double-edged sword effect of GenAI use on international students’ school adjustment

**DOI:** 10.3389/fpsyg.2026.1666169

**Published:** 2026-05-05

**Authors:** Huajun Ma, Xinglin Liu, Zimeng Chen, Tao Zhu, Qingnan You, Yinfei Zhao, Huijuan Li

**Affiliations:** 1 Research Center for the Regional Comprehensive Economic Partnership (RCEP), Member States of Ningbo University of Finance and Economics, Ningbo, China; 2 School of Economics, Shenzhen Polytechnic University, Shenzhen, China; 3 School of Management, Lanzhou University, Lanzhou, China; 4 School of Finance, Zhongnan University of Economics and Law, Wuhan, China; 5 School of Business Administration, Zhongnan University of Economics and Law, Wuhan, China; 6 College of Economics and Management, Hubei University of Arts and Science, Xiangyang, Hubei, China

**Keywords:** AI literacy, generative artificial intelligence, school adjustment, school membership, self-efficacy

## Abstract

Against the backdrop of increasingly frequent global educational exchanges, the issue of international students’ school adjustment has attracted much attention. The rise of Generative AI (GenAI) has brought new perspectives to research in this field, but existing studies have insufficiently explored the mechanisms by which it influences international students’ school adjustment. Based on the COR, this study collected 331 valid questionnaires from non-Chinese international students enrolled in three universities in North and South China through a three-stage time-lagged questionnaire survey to explore the double-edged sword effect of GenAI use on international students’ school adjustment. The results show that: on the one hand, GenAI use is associated with better school adjustment by positively predicting international students’ self-efficacy over a one-week lag; on the other hand, it is associated with poorer school adjustment by negatively correlating with their sense of school membership. Furthermore, AI literacy strengthens the positive path through which GenAI use affects school adjustment via self-efficacy, while buffering the negative path through which GenAI use affects school adjustment via school membership. This study not only helps explain the complex mechanism of GenAI use on international students’ school adjustment but also provides practical guidance for international student education management.

## 1 Introduction

With the acceleration of the internationalization of higher education, the scale of the international student population continues to expand. How to help international students achieve good school adjustment has become a focus in the field of education (Mao, 2025; Wray et al., 2025). China is currently the country with the largest number of overseas students in the world. According to data from the Ministry of Education of China, the number of Chinese students studying abroad exceeded 8 million in 2023, distributed in more than 100 countries and regions (Li et al., 2024). In the process of integrating into new academic systems and campus cultures, international students generally face multiple challenges such as difficulties in curriculum articulation, learning differences, and reconstruction of social circles (Gong et al., 2021). The level of school adjustment not only affects international students’ academic performance and quality of campus life but also relates to their future career development and the formation of intercultural competence (Sheng et al., 2022; Reynolds and Constantine, 2007). Although previous studies have explored the role of factors such as cultural intelligence, peer support, and institutional environment on school adjustment, the innovative development of Generative Artificial Intelligence (GenAI) has brought new perspectives to research in this field. As a cutting-edge application of artificial intelligence technology, GenAI, with its capabilities of intelligent interaction, knowledge generation, and personalized feedback, is gradually intervening in the learning and living scenarios of international students. However, the complex impact mechanism of GenAI on school adjustment has not been fully revealed, especially the ethical dimensions such as privacy security, algorithmic bias, and academic integrity associated with its use, which have received insufficient attention in existing studies (Biagini, 2025; Güneş and Kaban, 2025). Lo and Chan (2025a)further found obvious polarization in AI use perceptions between higher- and lower-GPA students in Hong Kong higher education, and ethical awareness and institutional detection effectiveness shape students’ responsible AI use. These ethical concerns may indirectly affect the establishment of international students’ self-efficacy and the formation of their sense of school membership by influencing their trust in technology, autonomous learning behaviors, and real interpersonal interactions, thereby interfering with the school adjustment process, while AI literacy (including ethical cognition) may moderate this effect. From a geographical perspective, the three research sites in this study cover China’s diverse educational landscape: one research-intensive university in northern China and two application-oriented financial and economic universities in southern China’s economic hubs, which shapes the application scenarios and impact pathways of GenAI for international students studying in China. This diversity shapes the application scenarios and impact pathways of GenAI (He and Liu, 2018). International students in China often rely on GenAI to navigate language barriers, such as understanding Mandarin course materials, and cultural norms specific to Chinese campuses, including group project dynamics and teacher-student communication protocols (Cao and Meng, 2022). Meanwhile, China’s collectivist cultural context places high value on interpersonal connections and school belonging. This cultural characteristic means that over-reliance on GenAI may carry greater risks of social disconnection compared to individualistic educational environments (Cao, 2025; Holmes and Zhgenti, 2024). This unique geographical and cultural setting underscores the need to examine how GenAI’s double-edged sword effect plays out in a context where academic success and social integration are deeply intertwined.

School adjustment, in a broad sense, is a dynamic process in which individuals mobilize and manage personal resources to meet environmental requirements in a new educational environment, mainly reflected in academic adjustment, interpersonal adjustment, and school identification (Baker and Siryk, 1989; Gao et al., 2023). The Conservation of Resources Theory (COR) states that individuals tend to acquire, maintain, and protect resources to resist stressful situations (Hobfoll, 2002), and GenAI can be regarded as a new type of digital resource that may play a dual role in international students’ school adjustment. On the positive side, GenAI helps international students quickly grasp key knowledge points and improve learning efficiency through functions such as curriculum content analysis, academic writing guidance, and study plan formulation (Chen, 2024), thereby enhancing their self-efficacy. Meanwhile, the psychological support and social companionship provided by GenAI based on emotion computing technology help international students alleviate campus loneliness and promote the formation of their sense of school membership. However, over-reliance on GenAI may also lead to the weakening of international students’ autonomous learning ability and the reduction of real interpersonal interaction opportunities, thereby having a negative impact on school adjustment. In addition, international students’ AI literacy may moderate the effect on school adjustment (Kong et al., 2023; Wang et al., 2023). Based on this, this study attempts to explore the complex impact mechanism of GenAI use on international students’ school adjustment based on the COR. The research results will deepen the understanding of the mechanism of international students’ school adjustment in the digital era and provide a scientific basis for universities to formulate intelligent education management strategies, with both theoretical innovation value and practical guiding significance.

## 2 Theory and hypotheses development

### 2.1 GenAI use, self-efficacy and school adjustment

Self-efficacy refers to an individual’s subjective judgment of their ability to successfully complete specific behaviors and achieve expected results (Bandura and Watts, 1996). In the process of international students adapting to new learning and living environments, the level of self-efficacy affects their attitudes and behaviors when facing challenges (Almukdad and Karadag, 2024). As an advanced digital tool, GenAI may enhance international students’ self-efficacy in the process of interacting with them. According to the COR, when individuals obtain resources that are conducive to their development, their confidence in their own abilities will be enhanced. GenAI has strong knowledge integration and generation capabilities. In terms of academics, it can provide international students with curriculum key point analysis, visual explanations of complex concepts, assist them in academic paper conception and writing, and help them break through language and knowledge bottlenecks (Qi et al., 2025). When international students successfully complete originally challenging learning tasks with the help of AI, they will intuitively feel the improvement of their abilities, thereby enhancing their academic self-efficacy. In campus life and social scenarios, GenAI can analyze campus culture and social norms, provide international students with communication strategy suggestions, and assist them in better participating in campus activities and integrating into social circles (Hsiao and Tang, 2025). International students with high self-efficacy will show stronger initiative and persistence when facing school adjustment challenges. They are more willing to actively explore campus resources, participate in academic and social activities, and respond to the pressure brought by academic difficulties and cultural differences with a positive attitude (Gebregergis et al., 2019; Almukdad and Karadag, 2024). This positive coping behavior pattern enables international students to more efficiently obtain the information and support needed for school adjustment, thereby improving their school adjustment level. Thus, this study proposes the following hypothesis:

*H1*: Self-efficacy plays a mediating role between GenAI use and international students' school adjustment.

### 2.2 GenAI use, school membership and school adjustment

School membership refers to an individual’s emotional and cognitive identification with their school, as well as the close connection established with the campus environment and the teacher-student group (Goodenow, 1993), which is an important psychological basis for international students to achieve good school adjustment. Although GenAI can provide convenience for international students, over-reliance on this technology is negatively associated with a sense of school membership, which in turn is positively associated with school adjustment.

According to the COR, if individuals over-rely on a single channel in the process of resource acquisition, it may lead to the loss of other important resources (Halbesleben et al., 2014). When international students take GenAI as the main resource acquisition channel, they will form a resource dependence inertia. This inertia is correlated with reduced investment in campus interpersonal interaction and potential loss of original social resources, which in turn shows a significant correlation with the attenuation of the important emotional resource of school membership. For example, frequent use of AI to complete course assignments makes international students lack opportunities for in-depth discussion on academic issues with teachers (Huang and Huang), weakening the knowledge transmission and emotional interaction between teachers and students; obtaining life information through AI instead of actively consulting classmates and seniors also hinders the establishment of friendships among peers. This reduction in interpersonal interaction is associated with difficulties in forming close emotional bonds with the campus environment and the teacher-student group, and a lower sense of belonging to the school (Xu, 2023; Huang and Huang, 2025). In addition, the information output of GenAI is often standardized and stylized, lacking the emotional warmth and personalized care in real interpersonal interactions. International students who rely on AI for information and emotional support for a long time will gradually get used to this “non-emotional interaction” mode (Nizamani et al., 2024), leading to limitations in the development of their emotional expression ability and social skills. When facing difficulties in studies and life, it is also more difficult for them to obtain effective emotional support and practical help from the campus environment, thus falling into a negative cycle in the adaptation process and reducing the overall level of school adjustment. Thus, this study proposes the following hypothesis:

*H2*: School membership plays a mediating role in the negative relationship between GenAI use and international students' school adjustment.

### 2.3 The moderating role of AI literacy

AI literacy refers to an individual’s ability to cognize, operate, and critically apply GenAI. International students with high AI literacy can systematically understand the functional boundaries, technical logic, and ethical norms of GenAI (Kong et al., 2023). Existing studies have shown that the level of AI literacy significantly affects users’ effectiveness and value judgment of intelligent technologies (Al-Abdullatif and Alsubaie, 2024). It is thus speculated that AI literacy will moderate the impact of GenAI use on international students’ psychology and behavior.

International students with high AI literacy can accurately identify the advantageous functions of GenAI, such as using its knowledge graph analysis ability to disassemble complex academic problems and optimizing the content quality of AI-assisted writing through prompt engineering (Gattupalli et al., 2023). This in-depth mastery of GenAI makes it easier for international students to obtain positive feedback on their academic tasks, thereby significantly enhancing their self-efficacy. At the same time, individuals with high AI literacy have stronger critical thinking skills in information, which can effectively avoid the wrong or biased content output by GenAI and reduce frustration in the learning process. International students with low AI literacy, due to their unfamiliarity with the operation logic and application scenarios of GenAI, find it difficult to transform it into an effective learning support tool. For example, the lack of prompt design skills leads to AI output content deviating from requirements, or the inability to distinguish the reliability of AI-generated information, thereby encountering more obstacles in learning (Bewersdorff et al., 2025). These inefficient use behaviors weaken the promoting effect of GenAI on academic performance and limit the improvement of self-efficacy. Therefore, AI literacy can strengthen the positive impact of GenAI use on self-efficacy, and the following hypothesis is proposed:

*H3a*: AI literacy positively moderates the relationship between GenAI use and self-efficacy, that is, the higher the level of AI literacy, the stronger the positive impact of GenAI use on self-efficacy.

According to the COR, an individual’s resource management strategy determines whether their resources are depleted or gained (Hobfoll, 2002). International students with high AI literacy regard GenAI as a supplementary tool rather than a substitute resource. While using it to complete learning tasks, they still maintain in-depth academic interactions with teachers and classmates and actively participate in campus social activities. They can reasonably plan the boundaries of AI use, avoid reducing interpersonal interactions due to over-reliance, and thus maintain a high sense of school membership. International students with low AI literacy are prone to falling into the “technology dependence trap” (Zhang and Pan, 2025), overusing GenAI to replace real interpersonal communication, such as completing course assignments through AI and reducing academic discussions with mentors, or relying on AI to recommend social plans and lacking active social behaviors. This improper use accelerates the loss of campus interpersonal resources (Martín Herrero et al., 2025) and exacerbates the sense of alienation between individuals and the campus environment. Therefore, AI literacy can alleviate the negative impact of GenAI use on school membership, and the following hypothesis is proposed:

*H4a*: AI literacy negatively moderates the relationship between GenAI use and school membership, that is, the higher the level of AI literacy, the weaker the negative impact of GenAI use on school membership.

Based on the COR and the previous hypotheses, this study further proposes that AI literacy will also moderate the mediating role of self-efficacy and school membership between GenAI use and school adjustment. Specifically, international students with higher AI literacy can use GenAI more efficiently. In this process, they can obtain more effective resources through GenAI, thereby improving their self-efficacy, and because they can reasonably grasp the boundaries of GenAI use, the impact of self-efficacy on school adjustment will be higher. At the same time, international students with higher AI literacy can avoid over-reliance when using GenAI, maintain a good sense of school membership, and thus weaken the impact of school membership on school adjustment; international students with lower AI literacy are prone to reducing their sense of school membership due to over-reliance on GenAI, thereby exacerbating the adverse impact on school adjustment. To sum up, this study further proposes the following hypotheses:

*H3b*: AI literacy positively moderates the mediating role of self-efficacy between GenAI use and school adjustment, that is, the higher the level of AI literacy, the stronger the indirect effect.

*H4b*: AI literacy negatively moderates the mediating role of school membership between GenAI use and school adjustment, that is, the higher the level of AI literacy, the weaker the indirect effect.

**Figure 1:**
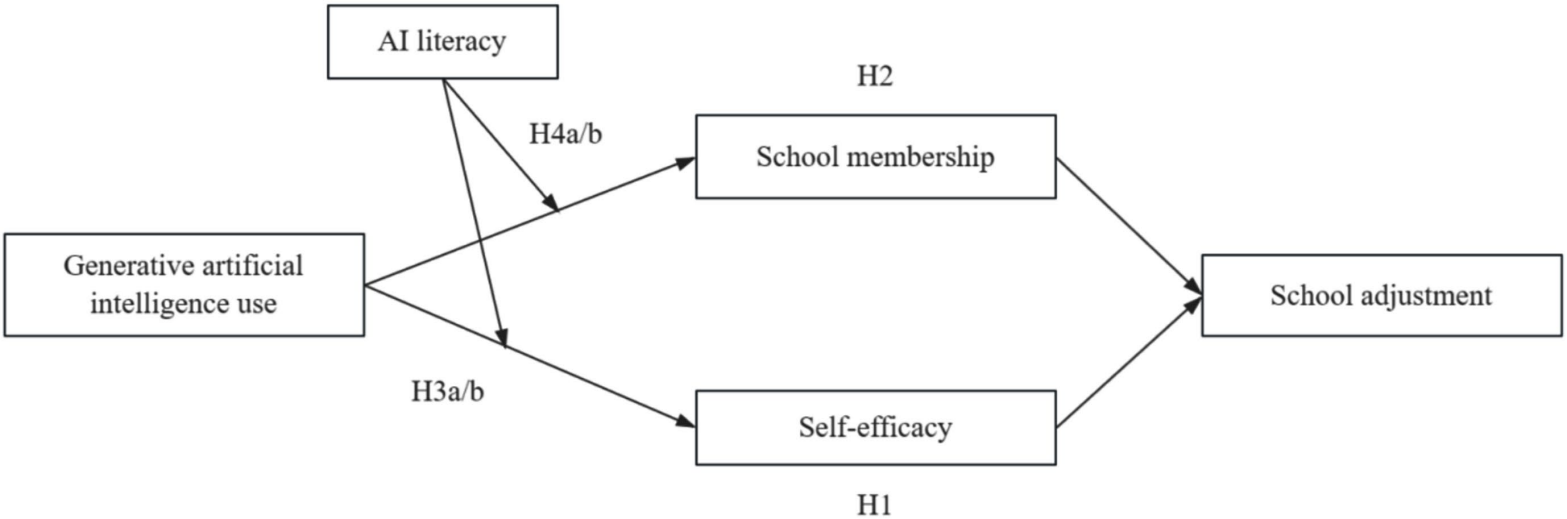
Theoretical model.

The corresponding theoretical model is shown in Figure 1.

## 3 Materials and methods

### 3.1 Participants and procedures

This study selected non-Chinese international students from three universities in China as the research sample, and conducted a voluntary online questionnaire survey for this group. Prior to data collection, all potential participants were provided with a detailed informed consent form. This form clearly outlined the study‘s purpose, procedures, the voluntary and anonymous nature of participation, potential risks and benefits, data confidentiality measures, and the right to withdraw at any time without penalty. Only those who provided explicit digital consent proceeded to the survey. The recruitment of participants was conducted through the international student affairs offices of the three selected universities. Full-time counselors responsible for international student management in each university acted as information transmitters only: they forwarded the voluntary survey notice (including research purpose, anonymity guarantee, incentive rules) to international students via official WeChat groups/email, and did not participate in any follow-up questionnaire filling process. No form of compulsion or inducement was used in the recruitment process, and counselors did not track or remind participants to fill in the questionnaire, which completely avoided undue influence on participants’ willingness to participate. All participants voluntarily clicked the questionnaire link in the notice to join the survey, and the research team had no direct contact with participants before the survey. To protect the privacy of participants, the questionnaires were filled out anonymously. To avoid common method variance, this study adopted a three-stage longitudinal data collection method. Questionnaires were distributed at different time points within 2 months, with an interval of 1 week between distributions. Before distributing the questionnaires, communication was conducted with participants to clarify the purpose of this study, and questionnaires were only distributed to international students who volunteered to participate. Questionnaires were distributed to participants via the Wenjuanxing platform by counselors. To improve the follow-up rate of the three-stage survey, a phased cash incentive mechanism was adopted: participants who completed the Time 1 questionnaire received a 5-yuan WeChat red envelope reward; those who completed both Time 1 and Time 2 questionnaires received an additional 5-yuan reward; and those who completed all three stages of the questionnaire and passed the validity check (no random answering) received a total 15-yuan WeChat red envelope reward. All rewards were issued by the research team through the questionnaire platform within 24 h after each stage of the survey was completed. Finally, matching was conducted based on the unique ID numbers registered by participants on the questionnaire platform for the three-time lagged survey. Specifically, at Time 1, control variables (gender, age, education level, major, study abroad duration, number of local friends), generative AI use, and AI literacy-related variables were collected. A total of 400 questionnaires were distributed, and 376 valid questionnaires were recovered; at Time 2, based on the recovered questionnaires from the previous round, questionnaires were distributed to collect self-efficacy-related variables, and 353 valid questionnaires were received; at Time 3, based on the recovered questionnaires from the previous round, questionnaires were distributed to collect international students’ school membership and school adjustment variables, and 331 valid questionnaires were received. The overall attrition rate was 11.97%, mainly attributed to natural attrition without systematic bias such as academic conflicts and voluntary withdrawal, which falls within the acceptable range for longitudinal research.

Among the 331 questionnaires, 186 were aged 18–22 (56.2%), 95 were aged 23–26 (28.7%), 45 were aged 27–30 (13.6%), and 5 were over 31 (1.5%). There were 168 males, accounting for 50.8%, and 163 females, accounting for 49.2%. In terms of education level, 206 were undergraduates (62.2%), 120 were postgraduates (36.3%), and 5 were doctoral students (1.5%). In terms of majors, 73 were in science and engineering (22.0%), 110 were in humanities and social sciences (33.2%), and 148 were in business (44.7%). In terms of study abroad duration, 138 were 1–6 months (41.7%), 97 were 7–12 months (29.3%), 75 were 13–24 months (22.7%), and 21 were over 24 months (6.3%); in terms of the number of local friends, 98 had 0 (29.6%), 135 had 1–3 (40.8%), 78 had 4–6 (23.6%), and 20 had more than 7 (6.0%). In addition, 217 were Asian students (65.6%), 42 were European students (12.7%), 51 were African students (15.4%), 14 were American students (4.2%), and 7 were Oceanian students (2.1%). In terms of Chinese proficiency (HSK), 89 had no HSK certificate (26.9%), 124 held HSK Level 1–3 (37.5%), 96 held HSK Level 4–6 (29.0%), and 22 held HSK Level 6 or above (6.6%). In terms of English proficiency, 287 had intermediate or above English proficiency (86.7%, able to communicate in English fluently), and 44 had basic English proficiency (13.3%, relying on translation tools for communication). All participants had at least basic communication ability in either Chinese or English, which guaranteed the validity of questionnaire filling.

All scales originally compiled in English were independently translated into Chinese by two bilingual researchers, whose academic backgrounds cover educational psychology and cross-cultural communication. Subsequently, a native English-speaking scholar with research experience in cross-cultural adaptation conducted a back-translation of the Chinese version back into English. The original English scales and the back-translated English version were carefully compared and revised to ensure strict semantic consistency and eliminate potential translation biases. The final questionnaire was designed with both Chinese and English versions, and participants could select the appropriate language version based on their own linguistic proficiency.

### 3.2 Measures

This study selected existing mature scales to measure relevant variables, using a 5-point Likert scale, with the measurement scale ranging from 1 (strongly disagree) to 5 (strongly agree).

GenAI: The scale used by Kraisila et al. was adopted (Kanont et al., 2024), with a total of 5 items, such as “Do you think you always use artificial intelligence to create text, images, or videos?” and “Do you think you will use artificial intelligence to support learning every time?” (*α* = 0.88).

Self-efficacy: The scale developed by Zhang and Schwarzer (1995) was adopted, with a total of 10 items, such as “I am confident that I could deal efficiently with unexpected events” and “I can solve most problems if I invest the necessary effort” (*α* = 0.91).

School Membership: The scale developed by Cheung and Hui was adopted (Cheung and Hui, 2003), with a total of 18 items, such as “I feel like a part of this school.” and “Most teachers in this school are interested in me.” (*α* = 0.91).

School adjustment: The measurement scale by Baker and Siryk was adopted, with a total of 19 items (Baker and Siryk, 1989), such as “I think I have the ability to solve my academic problems smoothly.” and “I am good at communicating with others by words.” (*α* = 0.87).

AI literacy: The scale by Lee and Park was adopted (Lee and Park, 2024), with a total of 25 items, such as “I have the ability to identify and solve technical problems of ChatGPT.” and “I can evaluate the accuracy of ChatGPT responses.” (α = 0.85).

Control variables: Age (1 = 18–22 years old, 2 = 23–26 years old, 3 = 27–30 years old, 4 = 31 years old and above), gender (1 = male, 2 = female), education level (1 = undergraduate, 2 = masters, 3 = doctoral), major (1 = science and engineering, 2 = humanities and social sciences, 3 = business), study abroad duration (1 = 1–6 months, 2 = 7–12 months, 3 = 13–24 months, 4 = more than 24 months), and number of local friends (1 = 0, 2 = 1–3, 3 = 4–6, 4 = more than 7) were used as control variables. Because previous studies have found (Cao and Meng, 2022) that these variables will affect the behavioral attitudes of international students, these variables were used as control variables to more accurately study the impact mechanism of generative AI use on the sociocultural adaptation of international students.

### 3.3 Common method variance

Although this study used a time-lagged two-stage data collection method, since the variable items are all self-reported, there may still be a certain degree of common method bias. Therefore, this study used the Unmeasured Latent Method Construct (ULMC) for testing (Richardson et al., 2009). The results showed that after putting all measurement items into the latent method factor, the six-factor structural model only had slight changes compared with the five-factor structural model (Δx^2^/df = 0.06; ΔCFI = 0.000; ΔTLI = –0.001; ΔRMSEA = 0.001; ΔSRMR = 0.013), and some fitting index results became worse, which means that adding a common method factor cannot significantly improve the fitting index. Therefore, there is no serious common method bias in this study.

### 3.4 Validity analysis

A structural equation model was constructed, and Mplus8.0 software was used to examine the discriminant validity of generative AI use, self-efficacy, school membership, school adjustment, and AL. Due to the large number of measurement items for variables, directly modeling with original items is prone to large parameter estimation bias (Little et al., 2002). Therefore, variables were randomly packaged in groups of three items. The specific test results are shown in Table 1. The results showed that χ^2^/df = 2.364, CFI = 0.963, TLI = 0.954, RMSEA = 0.052, SRMR = 0.047, indicating that the five-factor model has good validity.

**Table 1:** Results of the confirmatory factor analysis.

Model	Factors	χ^2^	*df*	χ^2^*/df*	CFI	TLI	RMSEA	SRMR
Model a	GenAI; SM; SE; SA; AL	723.56	306	2.364	0.963	0.954	0.052	0.047
Model b	GenAI+ SM; SE; SA; AL	1245.89	310	4.019	0.882	0.867	0.083	0.076
Model c	GenAI+ SM + SE; SA; AL	1987.22	313	6.349	0.795	0.781	0.105	0.098
Model d	GenAI+ SM + SE + SA; AL	3210.55	315	10.192	0.683	0.669	0.142	0.125
Model e	GenAI+ SM + SE + SA + AL	4120.33	316	13.039	0.502	0.487	0.215	0.189

GenAI: Generative artificial intelligence use, SM: School membership, SE: Self-efficacy, SA: School adjustment, AL: AI literacy.

Before hypothesis testing, to test the correlation between variables, the study used SPSS software for correlation analysis. The specific means, standard deviations, and correlation coefficients are shown in Table 2. GenAI had a significant correlation with school membership (*r* = −0.35, *p* < 0.001) and self-efficacy (*r* = 0.27, *p* < 0.01); school membership was positively correlated with school adjustment (*r* = 0.33, *p* < 0.001), and self-efficacy was positively correlated with school adjustment (*r* = 0.24, *p* < 0.01). The analysis results preliminarily supported the hypotheses.

**Table 2:** Mean, SD, correlations, and reliability.

Variables	1	2	3	4	5	6	7	8	9	10	11
1. Age	——										
2. Sex	0.05	——									
3. Edu	0.01	0.04	——								
4. Major	-0.01	0.02	0.05	——							
5. Time abroad	−0.03	0.07	0.01	0.04	——						
6. Local friends	0.15^*^	0.09	0.11	0.02	0.13^*^	——					
7. GenAI	0.07	0.14^*^	0.21^**^	0.03	0.02	0.01	**0.88**				
8. SM	0.05	0.06	0.13^*^	−0.05	0.04	0.12^*^	−0.35^***^	**0.91**			
9. SE	−0.10	−0.05	0.04	0.06	0.09	−0.03	0.27^**^	0.09	**0.91**		
10. SA	−0.08	0.12	0.07	−0.09	−0.07^*^	0.12^*^	0.21^**^	0.33^***^	0.24^**^	**0.87**	
11. AL	0.04	0.03	0.09	0.01	0.02	0.11	0.06	0.07	0.12^*^	0.07	**0.85**
*M.*	1.81	3.08	1.64	1.12	2.49	1.87	3.19	4.13	3.42	2.16	3.51
*S. D.*	0.52	0.73	0.51	0.73	0.69	0.71	0.83	0.92	0.62	0.74	0.87

** Correlation is significant at the 0.01 level (two-tailed). *Correlation is significant at the 0.05 level (two-tailed). The bold values indicated the Cronbach’s alpha. GenAI: Generative artificial intelligence use, SM: School Membership, SE: Self-efficacy, SA: School Adjustment, AL: AI literacy.

## 4 Results

### 4.1 Mediating effects

Before testing the hypotheses, the overall fit of the structural model (including mediating and moderating paths) was evaluated using Mplus 8.0. The model fit indices were as follows: *χ^2^* = 897.42, df = 386, *χ^2^*/df = 2.325, CFI = 0.958, TLI = 0.951, RMSEA = 0.049 (95% CI = [0.045,0.053]), SRMR = 0.043. These indices meet the recommended cutoff criteria (*χ^2^*/df < 3, CFI/TLI > 0.90, RMSEA < 0.08, SRMR<0.08; Hu and Bentler, 1999), indicating a good fit of the structural model to the data. This study used Mplus8.0 to test the hypotheses, and the sorted analysis results are shown in Tables 3, 4. The analysis results showed that GenAI use was significantly negatively associated with school membership (*γ* = −0.25, *p* < 0.01); GenAI use significantly positively predicted self-efficacy (*γ* = 0.21, *p* < 0.01). In addition, school membership was significantly positively associated with school adjustment (*β* = 0.37, *p* < 0.01), and self-efficacy had a significant positive effect on school adjustment (*β* = 0.31, *p* < 0.01). The Bootstrap method test found that the indirect associative effect of GenAI use on international students’ school adjustment through school membership was −0.079, S.E. = 0.01, 95% CI = [−0.126,-0.051]. The mediating effect value of GenAI use on international students’ school adjustment through self-efficacy was 0.083, S.E. = 0.02, 95% CI = [0.024,0.142], and H1 and H2 were verified.

**Table 3:** Results of path coefficient.

Variables	SM			SE			SA		
	Estimate	S.E.	*P*	Estimate	S.E.	*P*	Estimate	S.E.	*P*
Age	−0.03	0.01	0.072	−0.03	0.02	0.125	−0.01	0.03	0.092
Sex	0.04	0.04	0.058	0.00	0.08	0.253	0.02	0.05	0.095
Edu	0.02	0.02	0.064	0.04	0.01	0.071	0.03^*^	0.02	0.047
Major	0.13^*^	0.09	0.027	0.17^*^	0.08	0.020	0.05^*^	0.03	0.029
Time abroad	−0.11^*^	0.11	0.024	−0.05	0.09	0.085	0.04	0.04	0.067
Local friends	0.16^*^	0.03	0.017	0.08	0.02	0.079	0.12^*^	0.03	0.011
Independent variable									
GenAI	−0.25^**^	0.13	0.006	0.21^**^	0.14	0.008	0.23^*^	0.07	0.012
Mediator variables									
SM							0.37^**^	0.15	0.007
SE							0.31^*^	0.11	0.009
Moderator variable									
AL	0.19^*^	0.08	0.024	0.09	0.24	0.079	0.07	0.12	0.095
Interaction									
GenAI × AL	0.28^***^	0.04	0.000	0.25^**^	0.07	0.006	0.14^**^	0.12	0.010
Residual variance	0.68^***^	0.05	0.000	0.41^***^	0.02	0.000	0.73^***^	0.05	0.000
R2	26%			23%			37%		

***P < 0.001, **p < 0.01, *p < 0.05. GenAI: Generative artificial intelligence use, SM: School Membership, SE: Self-efficacy, SA: School Adjustment, AL: AI literacy.

**Table 4:** Results of mediating effect analysis.

Mediating effect	Estimate	S. E.	95%CI
GenAI → School membership → School adjustment	−0.079	0.01	[−0.126,-0.051]
GenAI → Self-efficacy → School adjustment	0.083	0.02	[0.024,0.142]

### 4.2 Moderation analysis

**Figure 2:**
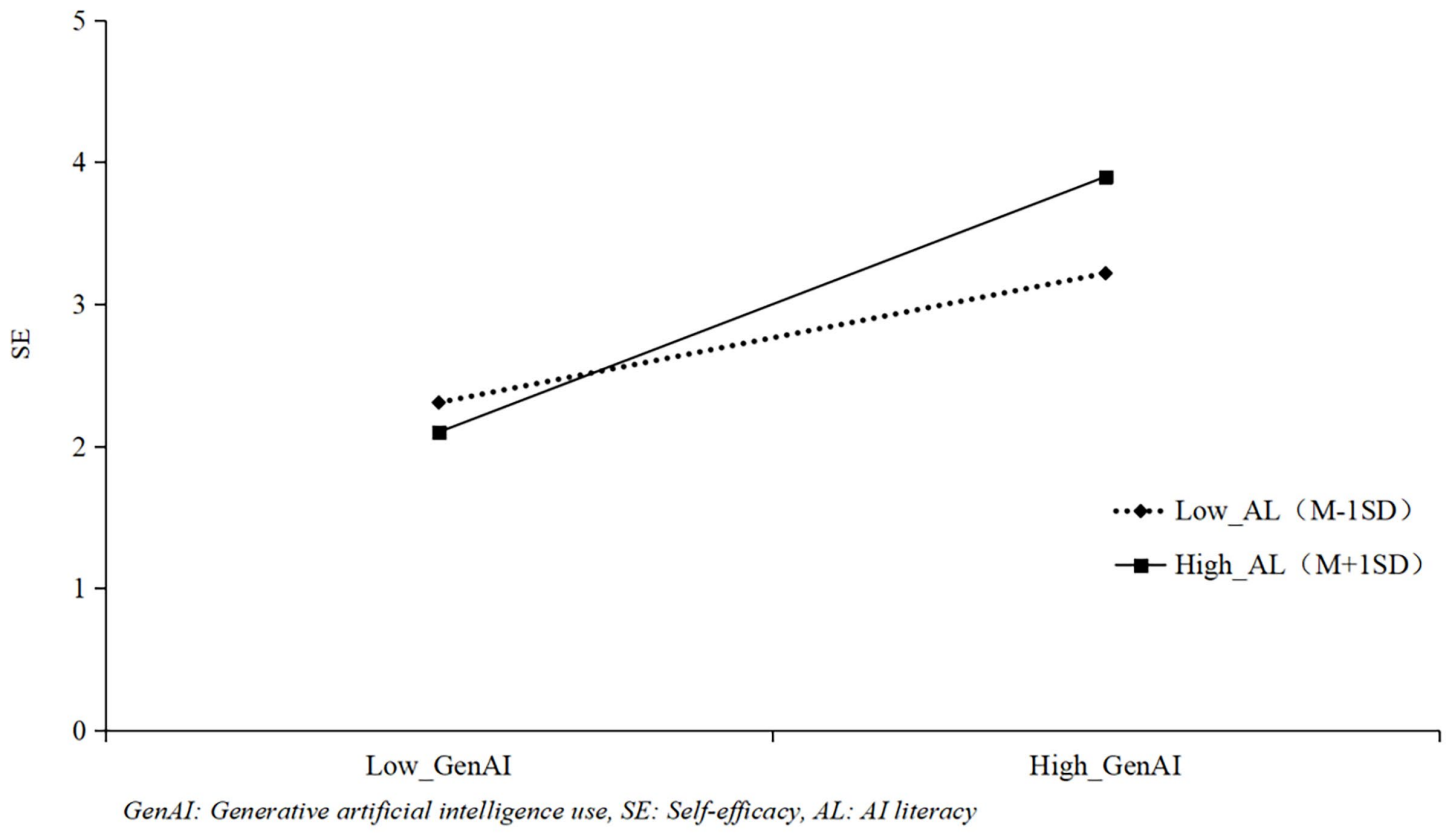
The moderating role of AI literacy between GenAI and Self-efficacy.

**Figure 3:**
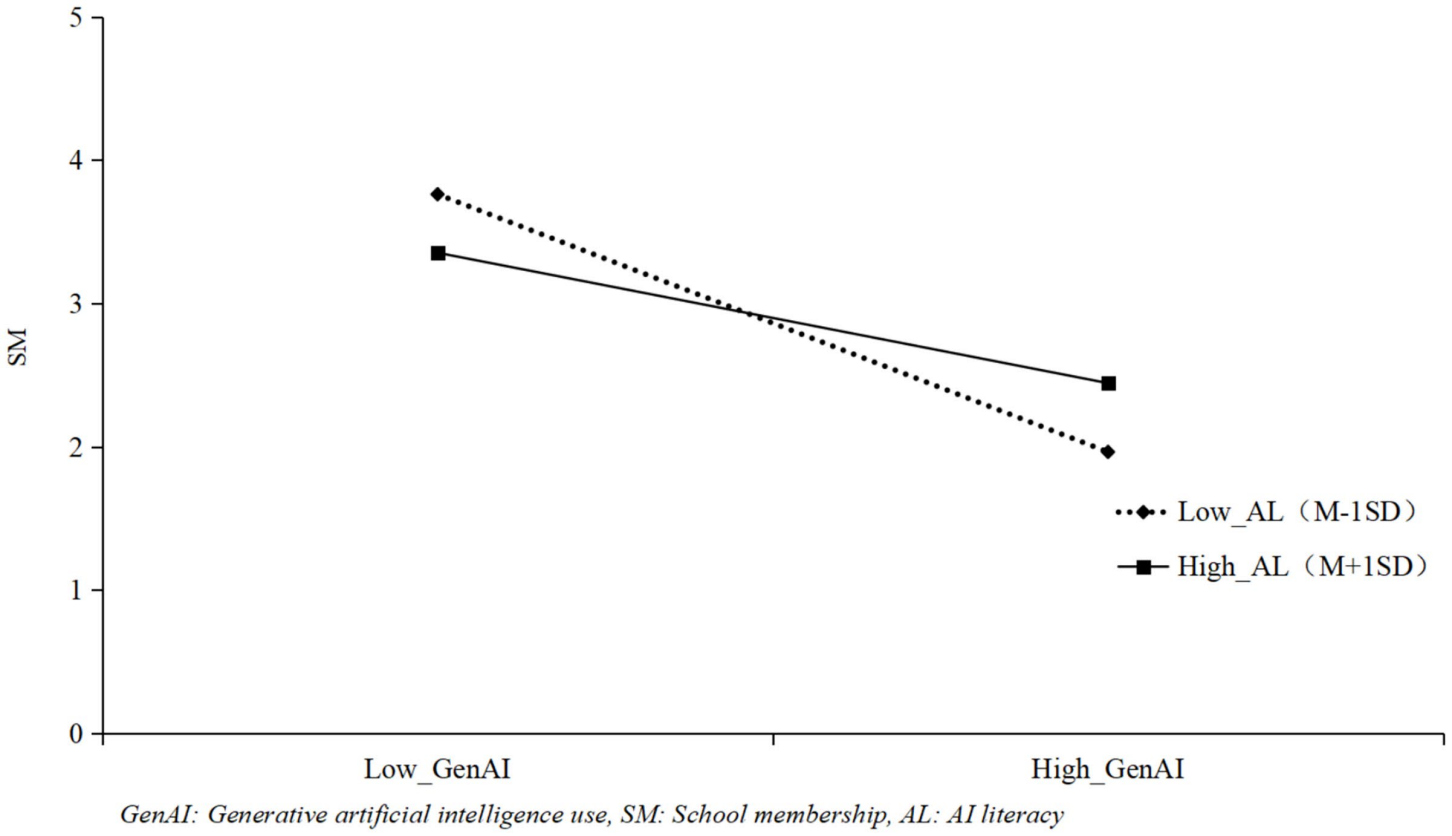
The moderating role of AI literacy between GenAI and school membership.

To test the moderating effects, the interaction terms (GenAI × AL) were constructed by mean-centering both GenAI use and AL scores first to avoid multicollinearity. In Mplus 8.0, the interaction effects were estimated using the product indicator approach (Little et al., 2002), as this method is compatible with the parceling strategy adopted for latent variable modeling. All latent variables were treated as continuous latent constructs with parceled indicators, and the interaction terms were included as observed variables derived from the centered main effect variables. Meanwhile, according to the analysis results in Table 3, the interaction term between GenAI use and AL was positively correlated with self-efficacy (*γ = 0.25, p < 0.01*), indicating that AL can positively moderate the linear relationship between GenAI use and self-efficacy, and H3a was verified. Drawing the moderating effect diagram (Figure 2) found that under the condition of high AL, GenAI use had a stronger impact on self-efficacy; conversely, under the condition of low AL, GenAI use had a weaker impact on self-efficacy. The effect of the interaction term between GenAI use and AL on school membership was significant (γ = 0.28, *p* < 0.001), indicating that AL can alleviate the negative impact of GenAI use on school membership, and H4a was supported. The moderating effect is shown in Figure 3. When international students have high AL, the negative impact of GenAI use on school membership is weaker; conversely, when international students have low AL, the negative impact of GenAI use on school membership is stronger.

### 4.3 The moderated mediation effects

Furthermore, to test the moderated mediation results, this study used Mplus8.0 software for 5,000 Bootstrap samples to analyze the moderated mediating effect. As can be seen from the analysis results in Table 5, under the condition of high AL, the mediating effect value of GenAI use on school adjustment through school membership was −0.067, 95%CI = [−0.109,−0.016]; under the condition of low AL, the mediating effect value of GenAI use on school adjustment through school membership was −0.118, 95%CI = [−0.162, −0.057]. The difference value of the mediating effects between the two was 0.051, 95%CI = [0.007,0.108], indicating that AL had a significant moderating effect on the mediating effect of school membership between GenAI use and school adjustment, and H4b was supported. Similarly, under the condition of high AL, the mediating effect value of GenAI use on school adjustment through self-efficacy was 0.089, 95%CI = [0.039,0.113]; under the condition of low AL literacy, the mediating effect value of GenAI use on school adjustment through self-efficacy was 0.025, 95%CI = [0.005,0.076]. The difference value of the mediating effects between the two was 0.064, 95%CI = [0.007,0.109], indicating that AL had a significant moderating effect on the mediating effect of self-efficacy between GenAI use and school adjustment, and H3b was supported. Based on the empirical analysis results of the study, all 6 proposed hypotheses (H1-H4b) have been fully verified.

**Table 5:** Results of the moderated mediation.

Moderator variable AI literacy	GenAI → School membership → School adjustment			GenAI → Self-efficacy → School adjustment		
	Estimate	S.E.	95% CI	Estimate	S.E.	95% CI
High (mean + 1SD)	−0.067	0.024	[−0.109, -0.016]	0.089	0.017	[0.039, 0.113]
Low (mean – 1 SD)	−0.118	0.023	[−0.162, -0.057]	0.025	0.014	[0.005, 0.076]
High vs. Low	0.051	0.024	[0.007, 0.108]	0.064	0.026	[0.007, 0.109]

### 4.4 Curvilinear effect analysis

To test the curvilinear effect of GenAI use on self-efficacy and school membership, a quadratic term of GenAI use was added to the baseline model, with AL and control variables still included as covariates. The results showed that the quadratic term of GenAI use had a non-significant effect on self-efficacy (*γ* = 0.08, *p* > 0.05), indicating no significant curvilinear relationship between GenAI use and self-efficacy. In contrast, the quadratic term of GenAI use had a significant positive effect on school membership (*γ = 0.22, p < 0.01*), which confirmed the inverted U-shaped curvilinear relationship between GenAI use and school membership: moderate use of GenAI has a weak negative association with school membership, while excessive use of GenAI leads to a more significant decrease in school membership. This result further supports the “double-edged sword” thesis of GenAI use, that is, the negative impact of GenAI on school membership is amplified with the increase of its use intensity.

## 5 Discussion

The theoretical contributions of this study are as follows: First, it enriches the technical perspective of research on international students’ cross-cultural adaptation. Previous studies have mostly focused on the impact of non-technical factors such as cultural intelligence and peer support on international students’ school adjustment (Xiaoying et al., 2023; San and Guo, 2023; Erturk and Luu, 2022), while this study incorporates GenAI, an emerging digital tool, into the analytical framework. Based on the COR, it reveals the role mechanism of technical factors in the adaptation process of international students, expands the theoretical boundary of cross-cultural adaptation research, and provides a new theoretical perspective for understanding the adaptation problems of international students in the digital era. Second, it reveals the “double-edged sword” effect of generative AI on international students’ school adjustment, reconciling the contradictory cognition of technical impact. Existing studies mostly hold a single positive or negative attitude toward the role of technology in the education field (Cao et al., 2024; Öztekin, 2024). This study finds through empirical testing that GenAI can not only positively promote school adjustment by improving self-efficacy but also have a negative impact due to reducing school membership, confirming the complexity of its impact. This conclusion integrates the dual effects of technology use and provides a more comprehensive theoretical explanation for understanding the relationship between technology and individual adaptation. Finally, it clarifies the moderating role of AL and deepens the understanding of the boundary conditions of GenAI’s impact. This study finds that AL not only positively moderates the relationship between GenAI use and self-efficacy but also negatively moderates the relationship between GenAI use and school membership, and further affects the mediating effects of both. This result reveals the key role of individual technical literacy in the process of technology’s role, provides a theoretical basis for defining the conditions for technology to play an effective role, and enriches the research on the impact of individual differences in the context of technology use (Chee et al., 2024). In addition, it is important to clarify that the AL scale adopted in this study is relatively new. Although the scale has demonstrated satisfactory reliability in the sample of this study, international students may have significant differences in their exposure to AI technology, educational practice backgrounds, and linguistic environments. These differences may affect their understanding of the scale items. Therefore, a cautious attitude should be adopted when interpreting the moderating effect of AL observed in this study. Future research could further validate AL measurement tools in different countries, linguistic contexts, and educational settings, and explore whether culturally adapted or simplified versions of the scale can yield consistent results.

The practical implications of this study are as follows: First, guide international students to use GenAI rationally to amplify its positive effects. Universities can help international students master the methods of using GenAI to analyze course content and optimize academic writing through special guidance courses to improve their academic self-efficacy. For example, for international students with language barriers, they can be guided to use AI for visual explanation of course knowledge and paper grammar correction to enhance their learning confidence and thereby promote school adjustment (Zhao et al., 2024). In addition, universities can also adopt GenAI as an auxiliary tool for the formative assessment of academic writing (Lo and Chan, 2025b), while paying attention to the perceptual tensions between instructors and students regarding AI-based assessment. Second, be alert to over-reliance on GenAI and maintain international students’ sense of school membership. Universities should create opportunities for real interpersonal interaction by organizing offline academic seminars, cultural experience activities, etc., to prevent international students from reducing communication with teachers and students due to over-reliance on AI (Zhai et al., 2024). It is stipulated that important course assignments need to be completed in combination with group discussions to prevent AI use from squeezing social resources, so as to maintain international students’ emotional connection with the campus environment. Finally, systematically improve the AL of international students and optimize their technical use ability. Universities can incorporate AL into the training system of international students (Biagini, 2025), offering courses on AI tool operation, information screening, and ethical norms to help international students accurately identify the functional boundaries of AI and avoid inefficient use or wrong reliance due to improper operation. For example, case teaching can be used to show how to design effective prompts to obtain high-quality AI feedback, improving their ability to control AI.

## 6 Limitations and future research

Although this study reveals the double-edged sword effect and mechanism of GenAI use on international students’ school adjustment, it still has the following limitations: First, despite adopting a three-stage time-lagged questionnaire survey method and confirming the absence of severe common method bias through ULMC testing, school membership and school adjustment were measured at the same time point (T3), which weakens the claims about the temporal ordering and causal mediation between these two variables. The current results only verify the significant associative relationship between school membership and school adjustment, but cannot fully confirm the unidirectional causal effect of school membership on school adjustment. In addition, the research data still rely on self-reports from international students. Due to the convenience constraints of the survey scenario, cross-validation through multi-source data such as teacher evaluations or peer reviews was not possible, which may have led to measurements of some variables being affected by subjective cognitive biases. Future research could adopt mixed-methods longitudinal designs to address the reliance on self-reported and short-term data in the present study. Specifically, repeated survey measurements collected across multiple academic semesters could be combined with semi-structured interviews to examine how international students’ GenAI use, self-efficacy, and school membership evolve over time. While survey data would capture changes in perceived adjustment, interview data could provide contextualized accounts of how students integrate GenAI into learning and social interactions, thereby triangulating the proposed psychological mechanisms. Second, the samples in this study were only sourced from non-Chinese international students enrolled in three universities in North and South China. The geographical distribution and types of institutions in the sample are relatively limited, which may affect the generalizability of the research conclusions. Educational systems and cultural environments vary across countries and regions, and international students’ GenAI usage habits and school adjustment needs may also differ. Future research could expand the sample scope to include international students from different continents and various types of institutions (e.g., research universities and application-oriented colleges). In addition, cross-cultural comparative designs could further strengthen triangulation by testing the model across different national and institutional contexts. For example, comparing international students studying in East Asian and Western higher education systems would allow researchers to examine whether the dual effects of GenAI use and the moderating role of AL remain consistent across cultures or vary according to educational norms and technological environments. Such comparisons would enhance both the robustness and external validity of the findings. Finally, this study focuses on the short-term impact of GenAI use, while international students’ school adjustment is a dynamic process. Long-term use of GenAI may trigger more complex effects (e.g., continuous changes in autonomous learning abilities, solidification of social patterns, etc.). Future research could adopt a longitudinal study design to track international students’ GenAI usage behaviors and school adjustment trajectories over an extended period, exploring the evolutionary patterns of their relationship over time, and providing a basis for universities to formulate phased strategies for guiding AI use.

## 7 Conclusion

The main purpose of this study was to explore the double-edged sword effect of international students’ Generative AI (GenAI) use on school adjustment and to test the mediating roles of self-efficacy and school membership, as well as the moderating role of AL. A three-stage time-lagged questionnaire survey method was used, resulting in a total of 331 valid samples. The study’s findings indicate that GenAI use has a dual impact on international students’ school adjustment: on the one hand, it is positively associated with school adjustment by positively predicting self-efficacy over a one-week lag; on the other hand, it is negatively associated with school adjustment by negatively correlating with school membership. AL was found to positively moderate the mediating path through self-efficacy and negatively moderate the mediating path through school membership. The results of this study contribute to the understanding of the complex psychological mechanisms behind GenAI’s impact on international students’ school adjustment in the digital era and provide theoretical support and practical insights for universities to optimize international student education management and guide rational AI use.

## Data Availability

The original contributions presented in the study are included in the article/supplementary material, further inquiries can be directed to the corresponding author/s.
